# Evaluation of the Parameters and Conditions of Process in the Ethylbenzene Dehydrogenation with Application of Permselective Membranes to Enhance Styrene Yield

**DOI:** 10.1155/2016/4949183

**Published:** 2016-03-16

**Authors:** Paulo Jardel P. Araújo, Manuela Souza Leite, Teresa M. Kakuta Ravagnani

**Affiliations:** ^1^School of Petroleum Engineering, Tiradentes University, 300 Murilo Dantas Avenida, 49032-490 Aracaju, SE, Brazil; ^2^Institute for Research and Technology (ITP), Tiradentes University (UNIT), 300 Murilo Dantas Avenida, 49032-490 Aracaju, SE, Brazil; ^3^College of Chemical Engineering, State University of Campinas, 500 Albert Einstein Avenida, 13083-852 Campinas, SP, Brazil

## Abstract

Styrene is an important monomer in the manufacture of thermoplastic. Most of it is produced by the catalytic dehydrogenation of ethylbenzene. In this process that depends on reversible reactions, the yield is usually limited by the establishment of thermodynamic equilibrium in the reactor. The styrene yield can be increased by using a hybrid process, with reaction and separation simultaneously. It is proposed using permselective composite membrane to remove hydrogen and thus suppress the reverse and secondary reactions. This paper describes the simulation of a dehydrogenation process carried out in a tubular fixed-bed reactor wrapped in a permselective composite membrane. A mathematical model was developed, incorporating the various mass transport mechanisms found in each of the membrane layers and in the catalytic fixed bed. The effects of the reactor feed conditions (temperature, steam-to-oil ratio, and the weight hourly space velocity), the fixed-bed geometry (length, diameter, and volume), and the membrane geometry (thickness of the layers) on the styrene yield were analyzed. These variables were used to determine experimental conditions that favour the production of styrene. The simulation showed that an increase of 40.98% in the styrene yield, compared to a conventional fixed-bed process, could be obtained by wrapping the reactor in a permselective composite membrane.

## 1. Introduction

Styrene is an important intermediate product in the petrochemical industry. The commercial demand for styrene grows at 6% per year. This popularity is mainly due to its recyclable character, which is not shared by other thermoplastics. About 90% of the total production of styrene is based on the catalytic dehydrogenation of ethylbenzene (EB). This reaction is reversible and endothermic, and the maximum conversion of EB under real process conditions is limited by thermodynamic equilibrium to about 45%. The industrial process is conducted at a high temperature, usually between 550 and 650°C. This temperature must be carefully controlled to avoid thermal stress on the catalyst, which can cause irreversible damage and most importantly the loss of selectivity by the formation of coke [1–4]. The operating pressure is usually atmospheric or lower and a catalyst of Fe_2_O_3_, associated with other metallic oxides, is used [5–7].

To supply the high energy load consumed by the highly endothermic reaction of dehydrogenation, a great quantity of steam is used in the reactor feed. It was shown that increase in styrene production over that of the industrial conventional fixed-bed unit is achievable, if design, operating parameters, and catalysts are properly chosen [8–10].

Recently, with improvements in the catalyst composition, the ratio of steam to oil (EB) in the feed has fallen considerably, from 20 to 6. The kinetic model of the dehydrogenation of ethylbenzene used here is based on the presence of ten species, involving six stoichiometrically independent linear reactions. The values of the corresponding reaction rate constants were given by Abdalla and Elnashaie [11], who obtained them by the adjustment of experimental data. The reaction generating styrene is reversible. When the concentration of hydrogen in the reactor is decreased, the equilibrium will be pulled to the right, increasing the conversion of EB and the selectivity to styrene, since the rate of conversion to toluene will be slower. Therefore, for separating hydrogen, membrane reactor can help the thermodynamic equilibrium to shift favourably in dehydrogenation reactions [12–14]. The potential for enhancement of styrene yield by catalyst improvement appears to be limited due to the fact that the main bottleneck is related to the thermodynamic equilibrium. Therefore, great efforts are needed to improve the performance through process design modification, and membrane separation technology has been intensively investigated in both modeling and experimental studies [9, 12, 15–21].

This paper proposed the removal of hydrogen from the catalytic medium through a permselective composite membrane. The driving force for removal of hydrogen is a gradient of the chemical potential of hydrogen, between retentate and permeate sides. To create this gradient, a pressure drop was maintained across the membrane wall. It presents an analysis of the parameters that control the performance of the fixed-bed reactor, surrounded by a permselective membrane, in the dehydrogenation of EB. In a process simulation, the operation conditions, fixed-bed geometry, and membrane geometry are assessed. Such variables were used to optimize the yield of styrene which is related to the conversion and the selectivity to the primary reaction. The demand for higher conversion of ethylbenzene, high yield, and selectivity of the desired reaction products especially styrene had led to new ingenious configuration and design of reactors for the dehydrogenation process.

## 2. Mathematical Modeling

For the simulation, a computer program was developed in Fortran. The kinetic model mentioned above was used, in which the rate constants were multiplied by an empirical factor observed in a commercial styrene catalyst provided by Stid-Chemie AG, München, Germany. These constants represent the effective rather than the intrinsic kinetics, in that the influence of diffusion is built into the rate constants, according to a pseudo-homogeneous model of plug flow.

The configuration of the reactor consists in catalytic fixed bed filled with the commercial 3 mm styrene catalyst wrapped by a membrane, which is compound of a stainless steel macroporous support, the microporous layer, and a palladium thin film.

It is assumed that there is no resistance to heat transfer across the membrane around the reactor.

### 2.1. Axial Transport

On the reaction side the axial molar flow rate of species *i* is controlled by its reaction rate and its rate of transport through the membrane:

(1) $$\begin{array}{c} \frac{d{\dot{n}}_{i}^{R}}{dz}={\left.\;A^{R}\rho_{b}\sum_{j=1}^{n}v_{ij}r_{j}-2\pi R_{R}J_{i}\;\right|}_{R_{R}}. \end{array}$$

In adiabatic conditions, *dT*/*dz* can be calculated by the enthalpy changes of the system. The pressure gradient (*dP*/*dz*) through the catalytic bed is calculated by the Ergun equation. The axial profiles of concentration, temperature, and pressure through the fixed bed (reaction side) are obtained by integrating this equation by the fourth-order Runge-Kutta method.

### 2.2. Radial Transport

The radial mass transport involves different transport mechanisms for the various membrane regions.

#### 2.2.1. Stagnant Gas Film

It is supposed that a stagnant film of gas is formed next to the membrane wall, exhibiting multicomponent diffusion. Stefan-Maxwell's equation is used to describe the concentration profile of species across this stagnant film. Temperature and pressure are assumed to be constant within the stagnant film.

#### 2.2.2. Macroporous Support

The dusty gas model was used to describe the profile in the macroporous support. This model assumes multicomponent diffusion of the molecular and Knudsen types and also viscous flow contributions. Assuming that the mass transport through the macroporous support is not due to the pressure gradient but to the concentration gradient, the dusty gas model for an ideal gaseous mixture is given by

(2) $$\begin{array}{c} \frac{dy_{i}}{dr}=\frac{RT^{R}}{P}\left(\;\overset{n}{\underset{j=1}{\sum }}\frac{J_{j}y_{i}-y_{j}J_{i}}{\left(\varepsilon /\tau \right)D_{ij}^{R}}-\frac{J_{i}}{D_{iK}^{e}}\;\right), \end{array}$$

where *D* Knudsen's effective diffusion coefficient of Fuller's correlation was used to determine the binary diffusivity *D*_*ij*_^*R*^ for the components of the process.

#### 2.2.3. Microporous Layer

It is assumed that Knudsen's diffusion [22] controls the flux of the various species through the microporous layer.

#### 2.2.4. Palladium Film

As the palladium film is permeable only to hydrogen, the boundary condition [23] is applied to the other components in the membrane wall. To describe the transport of hydrogen through the palladium film, Sievert's law was used (3).

It is assumed that the membrane is inert and operates in a stationary state, so that *J*_*i*_ is constant through the various layers. After discretization by finite differences and imposing the limit conditions for the concentration of each species on the reaction side, the system of equations was solved by the generalized Newton-Raphson method:

(3) $$\begin{array}{c} J_{H_{2}}=\frac{D_{H_{2}}C_{0}}{P_{0}^{1/2}\ln\left(r_{\text{ex}}/r_{\text{in}}\right)}\frac{1}{r}\left[\;{\left(\;P\;\right)}_{m1}^{1/2}-{\left(\;P\;\right)}_{m2}^{1/2}\;\right]. \end{array}$$

## 3. Simulations and Results

The styrene yield (percent ethylbenzene converted to styrene) was used as a measure of the performance of the process. Such process was conducted by removal of the hydrogen produced in the primary reaction. This removal was established by pressure drop of 199 kPa applied across the membrane, from retentate to permeate side of the reactor. This way it was possible to determine the operating conditions and optimal configuration of the reactor and the membrane through simulations to assess their influence on the process. The other operating ranges adopted are in Table 1, as well as the ranges of variation adopted for the configuration of the system proposed.

Each range was chosen on the basis of data from industrial operation and the restrictions of the process. Data of a commercial catalyst were used as a reference for the catalytic properties. The membrane properties were based on probable values for the types of material included in the membrane (stainless steel with palladium). These properties are described in Table 2.

For comparison, a simulation of the proposed model was run for a conventional fixed bed operating under standard conditions of industrial process [2], resulting in a styrene yield of 52.76%.

### 3.1. Analysis of Operating Conditions

#### 3.1.1. Energy Available to the System

Among the process conditions analyzed, the temperature and amount of steam are the parameters that reflect the energy available to bring about the reaction. To analyze these, the system was simulated while they were varied within the ranges in Table 1, trying to meet the energy requirements for the process without degrading the equipment. The molar ratio (steam/EB) varies industrially between 6 and 12.

**Figure 1:**
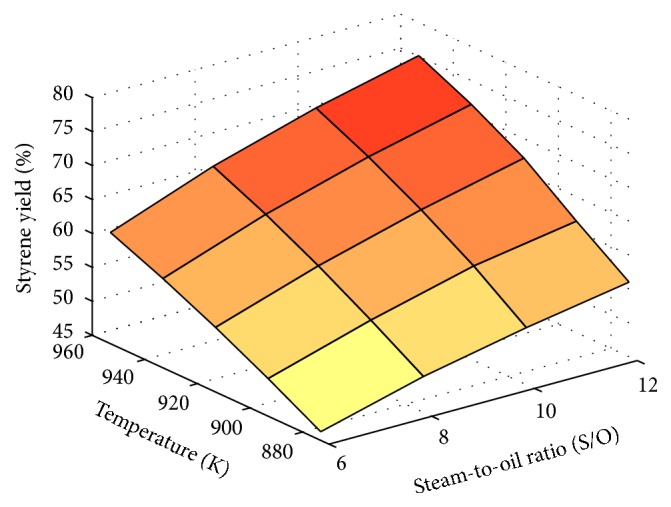
Influence of inlet temperature and steam-to-oil ratio on styrene yield.

The steam/EB ratio does not have to be the highest value tested in the analysis (Figure 1), but it (and the inlet temperature of the reactant) should be high enough to supply the energy needs of the reaction, and the ranges analyzed must not result in any damage to the equipment or to the products.

The best styrene yield was obtained at the highest values of temperature and steam-to-oil ratio tested, which were 953.15 K and 12. As a rise in temperature leads to an increased rate of hydrogen permeation through the permselective Pd membrane, the increased production of styrene at higher temperatures may be attributed to the removal of hydrogen from the reaction medium.

#### 3.1.2. Weight Hourly Space Velocity (WHSV)

To conclude the simulation of the influence of operating conditions on the system, a factor associated with the reaction velocity was analyzed, namely, the WHSV, over the range given in Table 1. The maximum yield was observed for the minimum space velocity (1.0 h^−1^). This behaviour was expected since this parameter is the ratio of the feed rate of reactants (kg/h) to the mass of catalyst (kg) in the reactor. This proportion increases when either the reactants flow more quickly or the catalyst mass decreases. In either case, there is reduced contact between the reactant and the catalyst, resulting in a lower styrene yield. Once the WHSV was fixed, the fixed-bed geometry became directly responsible for the flow of the reactor feed.

### 3.2. Analysis of the Fixed-Bed Geometry

To optimize the performance of the process, it is necessary to analyze the geometry of the fixed bed and the membrane that wraps it. In this section, the influence of the reactor length and the fixed-bed inner diameter will be examined.

#### 3.2.1. Inner Diameter × Reactor Length

As the radial dispersion in the fixed bed was not considered in this study, it was necessary to restrict this analysis to cases in which the reactor inner diameter is at least 8 (eight) times bigger than the diameter of the catalyst particle. The reactor used has a catalytic fixed bed filled with pellets of 3 mm (catalyst particle) in diameter of a catalyst commercial dehydrogenation of ethylbenzene. The fixed bed was filled with a permselective membrane and a thin layer of palladium.

**Figure 2:**
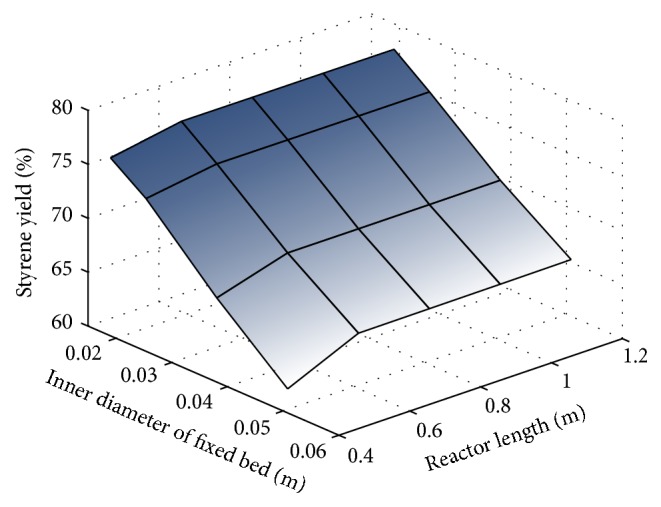
Analyses of reactor geometry.

In this simulation, the reactor inner diameters tested were those available on the market for the selected material (stainless steel), while the shortest possible reactor length was chosen because the material cost is decisive for the viability of a project. As can be seen in Figure 2, a smaller inner diameter results in a higher styrene yield, but since radial dispersion is neglected, one must choose the smallest diameter satisfying the above restriction (0.0254 m).

It was also observed that, in the range of reactor lengths analyzed, the styrene yield varies little, from 72.37% at 0.39 m to 72.71% at 1.2 m. This range of values is not sufficient to define an optimal reactor length. Therefore, it was decided to analyze the fixed-bed geometry at a constant reactant feed flow rate, instead of a predetermined space velocity.

#### 3.2.2. Analysis of the Reactor Volume at Constant Feed Rate

**Figure 3:**
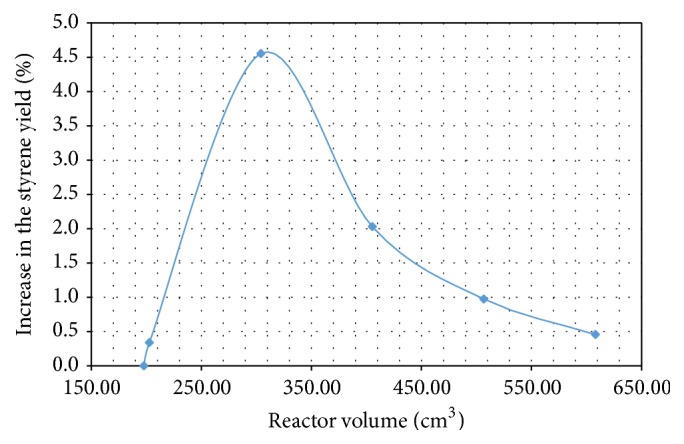
Influence of reactor volume on styrene yield at a constant mass flow.

This analysis was carried out with a constant reactor inner diameter of 0.0254 m, so that the reactor volume was varied by changing its length. Adopting a fixed mass flow at the entrance to the reactor of 9.08 × 10^−5 ^kg·s^−1^, determined from the data in Tables 1 and 2, simulations were run with reactor lengths from 0.4 to 1.2 m, corresponding to volumes from 197.62 to 608.05 cm^3^, and the styrene yield gain (relative to the shortest reactor) was calculated for each volume. According to Figure 3, the ideal reactor volume that satisfies the material restrictions on the process and minimizes the cost of implementation is 304.02 cm^3^. That represents a reactor length of 0.6 m for an inner diameter of 1^″^ (0.0254 m).

### 3.3. Analysis of Membrane Geometry

#### 3.3.1. Thickness of the Macroporous Layer

A thinner layer is expected to improve styrene yield. However, bearing in mind the membrane manufacturing techniques available on the market today, the tested range of thickness of the macroporous layer was selected from values available in the literature [24].

**Figure 4:**
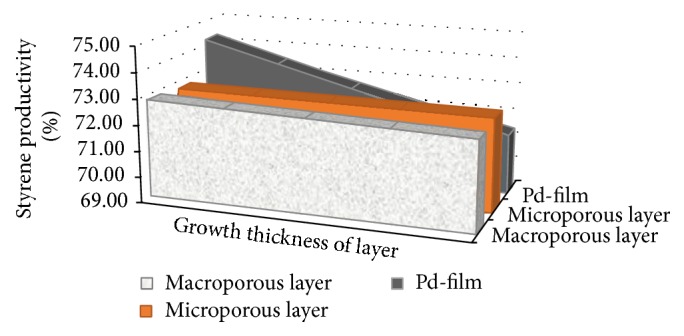
Influence of thickness of each membrane layer on styrene yield.

In the simulation presented in Figure 4, as expected, the best value of styrene yield was obtained (72.70%) with the thinnest macroporous layer. Its thickness (10^−3^ m) can be achieved by the technique of bimetallic multilayer deposition [25], without making the macroporous layer lose its capacity to support the membrane mechanically.

#### 3.3.2. Thickness of the Microporous Layer

Studies of permselective composite membranes usually fail to distinguish the thickness of the macroporous from that of the microporous layer, but as the latter affects directly the flux of species through the membrane, it was analyzed separately here.

The effect of the thickness of the microporous layer on yield was not significant. As it rose from 10 to 30 *μ*m (threefold thicker), styrene yield decreased almost linearly from 72.70 to 72.63%, as shown in Figure 4. As expected, the best styrene yield (72.7%) was achieved with the thinnest microporous layer, which allows a slightly greater flow of hydrogen through the membrane since there is a shorter route to permeate. Therefore, a 10 *μ*m thick microporous layer may be recommended for this system.

#### 3.3.3. Thickness of the Dense Metal Layer

In the transport of different species through the membrane, the stage with the greatest influence on hydrogen removal from the reaction medium is the dense metal layer. In dehydrogenation reactions, palladium is used in this layer, in view of its good permselectivity to hydrogen. The thickness of the palladium layer chosen for the simulation ranged from the thinnest (1.0 *μ*m) available with modern membrane manufacturing techniques [25] up to thicknesses already studied in the literature.

The simulation results indicate that the effect of reduction of the dense metal layer thickness in the range from 1.0 to 20.0 *μ*m (twenty times bigger) resulted in a styrene yield of 3% approximately. Hence, the thinner the palladium layer, the better the styrene yield. This is ideal for the application of palladium composite membranes in industrial processes, given the very high cost of this metal. These results reflect the fact that this layer imposes greater resistance to flow through the membrane.

Thus, in light of the simulation results and the restrictions on the manufacture of the membranes mentioned above, the dense metal layer thickness recommended for the best performance is 10 *μ*m.

### 3.4. Analysis of the Thickness of the Stagnant Gas Layer

**Figure 5:**
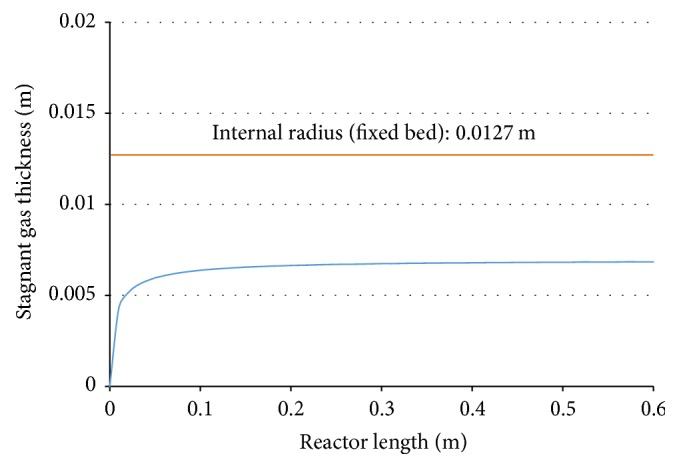
Profile of the stagnant gas layer in the fixed bed.

Besides analyzing the thickness of the membrane layers, it is important to consider the stagnant gas layer next to the membrane. The thickness of this stagnant gas layer depends on the fixed-bed geometry and the feed conditions (composition, temperature, and pressure). The proposed model assumes that, within the reactor bed, resistance to mass transfer in the radial direction can only be found in the stagnant gas layer. Thus, the internal radius of the fixed bed must be higher than the calculated thickness of the stagnant gas layer. As can be seen in Figure 5, nowhere does the thickness of the stagnant gas layer approach the internal radius of the fixed bed, so the application of the film theory is valid, showing a central region that allows gas to flow through the fixed bed (within the bulk gas phase) and a surrounding region where the flow meets resistance (stagnant gas).

### 3.5. Profile of Hydrogen through the Membrane

**Figure 6:**
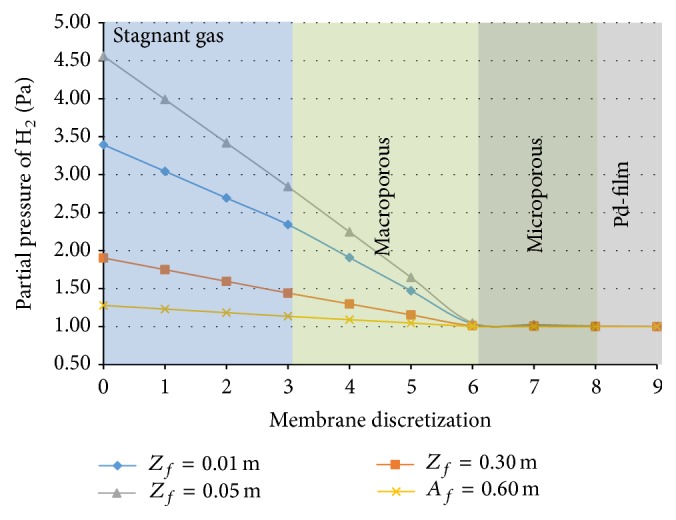
Profile of partial pressure of hydrogen in radial flow through the different layers.

The simulated radial profile of partial pressure of hydrogen through the layers around the reactor is plotted for various positions along the reactor in Figure 6. Note that the different membrane layers are not shown to scale (the Pd layer is 1,000 times thinner than the macroporous one).

The largest drop in the partial pressure of hydrogen is across the stagnant gas and macroporous layers. Such layers provide high rates of flow of the hydrogen, permeating the membrane.

Analyzing the difficulty of transport in the microporous layer, one can assume this is partly related to the pore diameter adopted in the simulation but mainly to the accumulation of components due to the phenomenon of polarization of the concentration next to this layer. As far as the transport through the palladium is concerned, although it is totally permeable only to hydrogen, this permeability is quite small. To allow an increase in this flux it would be necessary to reduce the resistance to flow imposed by the outer layers, which means altering their properties. However, within the existing restrictions on the forms of manufacture of the chosen material (stainless steel) and on the flux, imposed by the actual phenomenon of hydrogen transport through the Pd, the configuration adopted gives the best yield for this simulation.

## 4. Conclusion

Using the styrene yield as a criterion to assess system performance, the simulations carried out enabled both the operating conditions and the reactor and membrane dimensions that provided the best styrene yield to be determined.

By simulating the process with the recommended values for each parameter, the styrene yield was raised to 40.98% higher than the yield achieved with a conventional fixed bed (52.76%). This improvement demonstrates the importance of determining the favourable conditions for process development. Analyzing the hydrogen profile across the different layers of the membrane, it can be concluded that the microporous and palladium film layers prevent the further increase of the styrene yield, due mainly to the polarization of the hydrogen concentration next to the microporous layer. However this inconvenience cannot be overcome with the configuration adopted for the membrane, as current manufacturing techniques restrict the characteristics of these layers.

## Figures and Tables

**Table 1:** Ranges of variation of the operating conditions and system configuration.

Operating conditions	
Inlet temperature	873.15–953.15 K
Space velocity	1–1.6 h^−1^
Steam-to-oil ratio	6–12

Reactor configuration	

Length	0.39–1.2 m
Inner diameter	3/4′′–2′′
Equivalent diameter	5 mm

Membrane configuration	

Thickness (macro and micro)	1–3 and 10–30 mm
Thickness (Pd)	1–20 *µ*m

**Table 2:** Properties of the catalyst and membrane.

Properties of the catalyst	
Porosity	0.5
Density	2,150 kg·m^−3^
Diameter	3 mm

Properties of the membrane	

Porosity (macro and micro)	0.5
Tortuosity (macro and micro)	3
Pore diameter (macro)	0.2 *µ*m
